# The Michelangelo Effect: Art Improves the Performance in a Virtual Reality Task Developed for Upper Limb Neurorehabilitation

**DOI:** 10.3389/fpsyg.2020.611956

**Published:** 2021-01-07

**Authors:** Marco Iosa, Merve Aydin, Carolina Candelise, Natascia Coda, Giovanni Morone, Gabriella Antonucci, Franco Marinozzi, Fabiano Bini, Stefano Paolucci, Gaetano Tieri

**Affiliations:** ^1^Department of Psychology, Sapienza University of Rome, Rome, Italy; ^2^IRCCS Fondazione Santa Lucia, Rome, Italy; ^3^Department of Mechanical and Aerospace Engineering, Sapienza University of Rome, Rome, Italy; ^4^Virtual Reality Lab, Unitelma Sapienza, Rome, Italy

**Keywords:** virtual reality, art, psychophysics, stroke, rehabilitation, cognition, aesthetics, neuroscience

## Abstract

The vision of an art masterpiece is associated with brain arousal by neural processes occurring quite spontaneously in the viewer. This aesthetic experience may even elicit a response in the motor areas of the observers. In the neurorehabilitation of patients with stroke, art observation has been used for reducing psychological disorders, and creative art therapy for enhancing physical functions and cognitive abilities. Here, we developed a virtual reality task which allows patients, by moving their hand on a virtual canvas, to have the illusion of painting some art masterpieces, such as The Creation of Adam of Michelangelo or The birth of Venus of Botticelli. Twenty healthy subjects (experiment 1) and four patients with stroke (experiment 2) performed this task and a control one in which they simply colored the virtual canvas. Results from User Satisfaction Evaluation Questionnaire and the NASA Task Load Index highlighted an appropriate level of usability. Moreover, despite the motor task was the same for art and control stimuli, the art condition was performed by healthy subjects with shorter trajectories (*p* = 0.001) and with a lower perception of physical demand (*p* = 0.049). In experiment 2, only the patients treated with artistic stimuli showed a reduction in the erroneous movements performed orthogonally to the canvas (*p* < 0.05). This finding reminds the so-called Mozart effect that improves the performance of subjects when they listen to classic music. Thus, we called this improvement in the performance when interacting with an artistic stimulus as Michelangelo effect.

## Introduction

The human capacity to experience the beauty of things is particularly evident in the creation and appreciation of works of art. Experiencing the aesthetics of artworks is a very intriguing and controversial subject dealt with by philosophers and then by psychologists and neuroscientists (Di Dio et al., 2016). The processes involved in such a capacity include three different levels of aesthetic experience which have been evaluated and discussed: a perceptual, a cognitive and an emotional stage (Di Dio et al., 2016). It has opened a new field of research named neuroaesthetics (Zeki, 2002). Surprisingly, the aesthetic experience of artworks depicting both human subjects and nature scenes seems to involve also brain motor areas. Indeed, it has been demonstrated that the dynamic human figures seem to activate more precuneus, fusiform gyrus, and posterior temporal areas, with respect to nature scenes that activate more occipital and posterior parietal cortex, both involved in visuospatial exploration and pragmatic coding of movement, as well as central insula (Di Dio et al., 2016). Static nature paintings further activated central and posterior insula, probably because they evoke aesthetic processes requiring an additional proprioceptive and sensorimotor component implemented by “motor accessibility” to the represented scenario, which is needed to judge the aesthetic value of the observed painting (Di Dio et al., 2016). It is important to highlight that further results also showed the involvement of the cortical motor system even in the viewing of static abstract artworks (Umilta’ et al., 2012).

The sensorimotor networks activated by viewing an art masterpiece could be related to the recognition of emotions displayed by expressions of painted persons (Adolphs et al., 2000), to the mirror neuron networks activated by the actions performed by painted persons (Freedberg and Gallese, 2007), to the ideal possibility to walk in the scene (Di Dio et al., 2016), and even to the empathetic engagement activating simulation of the motor program that corresponds to the gesture implied by the trace done by the painter into the observer (Knoblich et al., 2002; Freedberg and Gallese, 2007).

Artworks would feed into a general feeling of pleasure, motivation, and arousal (Duckworth et al., 2014). Brain arousal and motivation are two fundamental aspects also in neurorehabilitation, together with active participation and treatment intensity (Paolucci et al., 2012). Based on these principles, music-therapy, for example, has been proposed in neurodegenerative diseases such as Parkinson’s disease (De Bartolo et al., 2020a) or stroke (Verna et al., 2020). Furthermore, it was observed that listening to Mozart music improves the performance of subjects during the execution of a task, and it was called “Mozart effect” (Victorino et al., 2020). If Music-therapy could be performed by listening or generating music, Art-therapy was often limited to asking patients to paint, and not to observe art masterpieces. Action-observation neural mechanisms, based on the activations of mirror neuron networks, have been exploited in rehabilitation showing to patients some videos, but it does not involve the above described wide brain activations. The ideal scenario for combining the potential sensorimotor benefits of painting and the wide brain arousal induced by art would be to require the patient to copy a masterpiece. Unfortunately, very few humans are able to do it, so it seems practically impossible for a patient with an affected upper limb. However, virtual reality technology may provide valid support in simulating this task. Furthermore, technological sensors may also provide reliable measures of the subject’s performance during the task (Iosa et al., 2016; Tieri et al., 2018).

Virtual reality (VR) is a new technology that can give the illusion to be in another place, thanks to the so-called sense of presence (Sanchez-Vives and Slater, 2005), and to respond in a realistic way to virtual stimuli, including both physiological (Meehan et al., 2002; Tieri et al., 2015; Fossataro et al., 2020) and neural reactivity (Vecchiato et al., 2015a,b; Pavone et al., 2016), the so-called sense of agency, and even to do impossible or uncommon things, living unusual experiences, in a safe and controlled situation (Tieri et al., 2015), as often occurs in immersive videogames. Virtual reality has been suggested also as a useful tool in neurorehabilitation of patients because it may increase motivation and enjoy during therapy process (Cho et al., 2013). Undoubtedly, technologically-assisted therapy should favor gaining maximum advantage from the opportunities provided by VR-technology for obtaining significant benefits in terms of rehabilitative outcomes (Tieri et al., 2018). Indeed, VR showed promising results in the therapy process thanks to interactive and direct training opportunities given to patients affected by neurodegenerative diseases, such as those with multiple sclerosis (Calabró et al., 2017). So, despite sometimes VR is used just to replicate the activities of daily living, it can provide the possibility to give the illusion to do something otherwise impossible, such as painting a masterpiece of the history of art. Even though the experience of standing in front of an authentic work of art cannot be replaced in terms of explicit hedonic attributed values by virtual reproductions, it has been shown as faithful high-quality virtual reproductions of artworks could be as arousing as the original works of art (Siri et al., 2018).

In the present study, we immersed healthy participants and a group of patients with stroke in a virtual environment where they had the illusion to paint famous masterpieces. Furthermore, during the task, their performance was assessed by measuring kinematic parameters related to the hand trajectory with respect to the virtual canvas. We also evaluated the acceptability and usability of this VR-system.

The main aim of this study was to validate the hypothesis that the performance of subjects could be improved when they interact with an art masterpiece, with respect to control stimuli, during the execution of the task performed in VR. This approach could open a novel way for rehabilitation programs for multiple users and can be helpful in administering to patients with stroke an art therapy for upper limb recovery.

## Materials and Methods

The study was divided in two experiments, one conducted on healthy subjects and one involving a group of four patients with stroke. The research protocol was designed in accordance with the 2013 Declaration of Helsinki and approved by the Ethics Committee of the Santa Lucia Foundation. Each volunteer provided written informed consent to participate in the study.

### Hardware and Software Equipment

Each subject sat wearing the Oculus Rift Head Mounted Display and taking into his/her hand (the preferred one for healthy subjects, the paretic one for patients with stroke) an Oculus Controller joystick which allowed to interact with the virtual stimuli. The virtual environment, designed by using 3ds MAX 2018 and implemented in Unity 2018 game engine software, consisted of a large and comfortable room (with a door, a window, two lamps, a sofa; Scale 1:1) in the middle of which there was a canvas on an easel. The subject could interact with the canvas with a virtual sphere, displayed in VR in the same place of the real hand, which could be controlled with the Oculus Controller by means of a customized script in C# (see Supplementary Video 1).

Each virtual canvas was 60 cm × 40 cm and appeared white at the beginning of the task. Subjects were instructed that the sphere can color the canvas when put in contact with it, forming a painting. The illusion is given thanks to a white thin virtual panel (composed by 19 × 13 = 247 pixels, pixel area: 10 cm^2^) placed in front of the canvas which occluded the visibility of an underlying image. When the subject touched the virtual panel with the sphere, the target pixels were automatically deleted allowing to see a part of the underlay picture. To overcome the missing tactile information concerning the real touch of the virtual canvas, we included visual feedback about the shadow of the virtual sphere on the canvas itself (in order to enhance the visual information about its position in the 3D-space) and also a change in the color of the sphere, from grey to green, when the virtual sphere touched the canvas colliding the panel’s pixels, or becoming red if an erroneous movement was performed beyond the canvas. The dimension of canvas and pixels were chosen according to preliminary tests involving other patients with stroke (not included in the present study). Before the experiment, each participant underwent to a calibration task. Then the participant was asked to color the entire canvas in the shorter time possible, but without missing any pixel. The performance of the subject was recorded through a customized C# script implemented in Unity which allowed to track and record in real-time the position of the virtual sphere/real hand in space. Each subject was also instructed to move their upper limb without moving the trunk. Each trial was controlled by a researcher who monitored what happened into the virtual environment on the computer’s monitor and by a physiotherapist who monitored the movements of the subjects, especially for avoiding trunk compensation strategies of patients. Each experimental session was composed of 10 trials, that could be 10 artistic paintings or 10 control stimuli, during which the participants painted the canvas. After each trial, the colored canvas disappeared and a new white canvas appeared on the easel.

**FIGURE 1:**
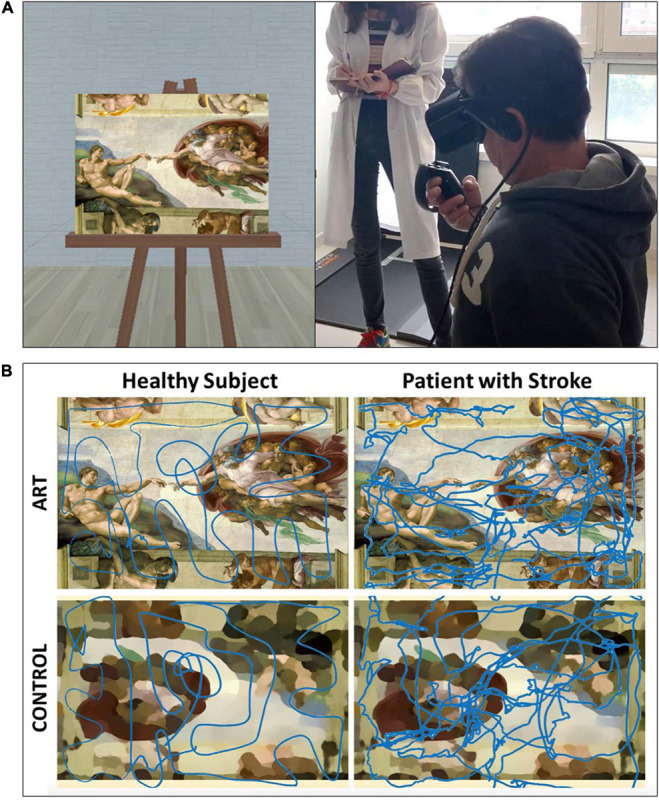
**(A)** Experimental setup; Left-side represents an example of the art masterpieces (the Creation of Adam of Michelangelo) presented during the task; Right-side shows a patient with the experimental setting of Oculus Headset and Controller, under the supervision of experimenter. **(B)** Example of an experimental stimulus of the art masterpieces (the Creation of Adam of Michelangelo) and the relevant control stimuli (below), with superimposed the hand trajectories for a healthy subject (on the left) and a patient (on the right).

The artistic paintings were chosen according to three criteria: to cover different époques (starting from Renaissance up to the twentieth century), to cover different styles (realism, baroque style, impressionism, post-impressionism, Ukiyo-e style, expressionism, and cubism), and especially to have similar proportions each other that could be matched by the size of the canvas. The following paintings were chosen: The Annunciation (Da Vinci, 1475), The Birth of Venus (Botticelli, 1485), The creation of Adam (Michelangelo, 1511), The Vocation of Saint Matthew (Caravaggio, 1600), The great wave of Kanagawa (Hokusai, 1831), Rowers Breakfast (Renoir, 1882), The bedroom (Van Gogh, 1888), The Night Café (Van Gogh, 1888), The Dance (Matisse, 1910), The Three Musicians (Picasso, 1921). For avoiding possible bias, the same colors and the same amount of brightness of the art masterpieces were maintained into the control stimuli; each masterpiece, was realized by blur-filter for control painting, and then reversing both left-right and up-down the image (GIMP software, Gnu Image Manipulation Program, version 2), as shown in Figure 1.

### Self and Instrumented Assessment

A crucial aspect of the present study was to assess the acceptability and usability of the implemented VR task. In fact, to gain valid and reliable data, it is fundamental to assess usability and mental workload of the used tools and methods. Thus, User Satisfaction Evaluation Questionnaire (USEQ) and Nasa Task Load Index (NASA-TLX) were administered to subjects after the execution of the VR session. Both scales test six domains of the self-perception about the usability and the perceived load demand of the tool. In particular, USEQ has six questions (for example: “Did you enjoy your experience with the system?”) with a five-point Likert Scale for each one of this item with a score going from 1 to 5, and hence a total score ranging from 6 (poor satisfaction) to 30 (excellent satisfaction). The six items test the self-perceived satisfaction, efficacy, efficiency, easiness-to-use, fatigue and self-perceived utility about the performed exercise. NASA-TLX has six questions with a ten point numerical rating scale for each one of the item (for example: “How physically demanding was the task?”) with a score ranging from 1 to 100. It tests the self-perceived mental demand, physical demand, time demand, effort, performance, and stress. For patients enrolled in the experiment 2, the participation at each session was assessed also using the Pittsburgh Rehabilitation Participation Scale (PRPS) (Lenze et al., 2004; Kokini et al., 2012). Patients were also clinically evaluated at baseline using the Fugl-Meyer scale, the Box and Blocks test, and 9-hole peg test. Fugl-Meyer assessment scale is designed to assess motor functioning, balance, sensitivity, proprioception and joint functioning in patients with post-stroke hemiplegia. It includes 63 items, each one using a 3-point ordinal scale (score: 0 inability, 1 deficit, 2 no deficit), with a maximum total score of 126. The Box and Block Test measures unilateral gross manual dexterity, counting the number of blocks the patient is able to move, one by one, from a compartment of a box to the other one, within 60 s. The Nine-Hole Peg Test is used to measure hand dexterity, by measuring the time need to take nine pegs from a container, one by one, and place them into the holes on a specific board having nine holes. For each trial, the subject’s performance was also quantitatively assessed starting from the spatio-temporal data of the joystick position with respect to the canvas. So, the following parameters have been computed: Time to Complete the Trial (TCT, from the moment in which the new white canvas appears to that in which the last pixel was colored), Length of Trajectory of the sphere on the canvas (LoT, evaluated in meters of the pathway performed on the frontal plane in which laid the canvas), root mean square of depth errors (RMSe, it was compute as the root mean square of the orthogonal distance of the sphere with respect to the canvas, considering the perfect contact as zero: it was a measure of erroneous movements performed along the axis orthogonal to the plane of the canvas).

### Experiment 1

In the Experiment 1, 20 healthy subjects were involved (mean age: 30.2 ± 7.1 years, 10 males and 10 females, without any neurological disease or orthopedic problems at the upper limb). Healthy participants performed in the same day two sessions: one with 10 paintings and one with 10 control stimuli. Half of subjects firstly saw the paintings and then the control stimuli, the reverse for the other half of participants. After each session, USEQ and NASA-TLX were administered to the subjects.

### Experiment 2

In the experiment 2, we enrolled 4 patients with stroke (3 males, 1 female, mean age: 59.5 ± 12.8 years, time from acute event longer than 3 months). Each patient performed 4 sessions of 10 stimuli each one, in 8 days. Two patients interacted with the same art-masterpieces of experiment 1 in all the four sessions while the other two patients interacted with the same control stimuli of experiment 1 in all the four sessions. All the patients had cognitive skills adequate to understand and to try to execute the task.

### Statistical Analysis

Data are reported in terms of mean and standard deviation. In the experiment 1, the kinematic continuous measures were compared using Repeated Measure Analysis of Variance (RM-Anova) using as the main factors art (vs. control stimuli), and paintings. Effect size was computed as the partial eta squared. Ordinal scale scores were compared using Wilcoxon test. In the experiment 2, for each patient the parameters recorded during the fourth session were compared to the relevant values computed during the first session by means of paired *t*-test. Data of experiment 2 were also used to compute the required sample size of a further randomized control trial with an enrolment ratio 1:1. The level of statistical significance was set at 5%, except for *post hoc* analysis performed applying the Bonferroni correction to the significance level.

## Results

### Experiment 1

The length of trajectory was significantly lower for artistic paintings vs. colored canvas (*p* = 0.001). This main effect was not statistically significant for TCT. As shown by the significant interaction, the time significantly depended by the type of painting. *Post hoc* analyses showed a significant longer time for The Annunciation of Leonardo with respect to its control stimulus (6.7 ± 2.7s vs. 4.4 ± 0.9s, *p* = 0.0025), whereas shorter time for The Bedroom of Van Gogh with respect to its control (4.8 ± 1.0s vs. 8.0 ± 3.7s, *p* = 0.0017), but it was mainly due to the differences between the two control stimuli than between the two artistic paintings (3.0 ± 3.2s vs. 1.4 ± 2.7s, *p* = 0.019).

The self-reported assessment showed a slight but significant difference in terms of the perceived physical demand related to the task (NASA-TLX, second domain). In fact, despite the canvas are equal in dimension, a lower physical demand was perceived when subjects interact with paintings with respect to control stimuli (22.1 ± 21.7% vs. 27.1 ± 18.9% of the maximum load, *p* = 0.049). No significant differences were found for the other domains of NASA-TLX, nor for those of USEQ.

### Experiment 2

Patients provided similar NASA-TLX and USEQ scores for both the conditions (art and control stimuli). They reported a mean score of 4.75 (on 5) about success in using the device, the minimum score of 1 for discomfort, and the highest score for the other domains of USEQ. Similarly, the mean scores for NASA-TLX were 9% for mental demand, 6% for physical demand, 4% for temporal demand, 93% for self-assessed performance, 5% for effort, 1% for frustration. The average score of Pittsburgh Rehabilitation Participation Scale ranged from 5.25 to 6 among the four sessions.

**FIGURE 2:**
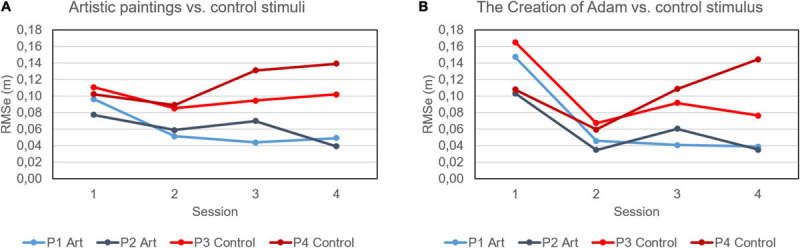
Root mean square of hand position along the axis orthogonal to canvas (RMSe). **(A)** the average values of the 10 stimuli for each one of the four patients along the four sessions; **(B)** the RMSe computed for The Creation of Adam of Michelangelo (in P1 and P2) and the relevant control stimuli (in P3 and P4). In both panels, the patients treated with artistic stimuli were P1 (light blue) and P2 (dark blue), whereas those with control stimuli P3 (light red) and P4 (dark red).

Table 2 shows the baseline clinical assessment and the comparison of the first vs. fourth session for each patient. At the first session, patients showed a longer time to complete the task (TCT) and higher errors (RMSe) with respect to healthy subjects of experiment 1, as expected. Patients treated with artistic stimuli showed significant improvements for all the three computed parameters, especially halving the RMSe. Conversely, one patient treated with control stimuli just showed a significant reduction of length of trajectory, but an increment of RMSe. This is a parameter related to erroneous movement along the axis orthogonal to the canvas, and Figure 2 shows the values of this parameter for each patient in terms of average values among all the paintings (on the left), and also the specific trend for the Creation of Adam of Michelangelo, for which this effect was magnified (on the right).

Using the data of the length of trajectory in sessions 4 as pilot data to design a randomized controlled trial with 10 sessions for each patient, the required sample size to obtain statistically significant results in terms of length of trajectory was 10 patients for each one of the two groups.

## Discussion

In the present study, we capitalized on the power of immersive virtual reality to induce the illusion to paint famous masterpieces in order to evaluate whether the motor performance of healthy subjects and neurological patients could be affected by the observed masterpiece vs. a simple control canvas and whether the proposed VR task reaches a good level of acceptability and usability. In particular, based on previous evidences provided by neuroesthetic studies, we tested the hypothesis that the art masterpieces can improve the performance of subjects during virtual painting.

In general, USEQ and NASA-TLX scores obtained supported the idea of a good level of usability of the developed VR-system for both healthy subjects and patients, suggesting that this approach can be promising for future development of VR-based rehabilitative task.

Furthermore, kinematics analysis related to the performances suggested that the art masterpieces positively affect the execution of the exercises. Indeed, the results of experiment 1 showed that healthy subjects completed the task with shorter hand pathways and lower perception of physical demand when an art masterpiece appearing on the canvas. On the other side, in the experiment 2, only the two patients who interacted with art masterpieces showed significant improvements in all the three computed parameters as compared with the two patients who performed the task with the control stimuli, especially the reduction of errors orthogonal to the canvas, as also highlighted in Figure 2 for the painting “The Creation of Adam” of Michelangelo.

These findings of paintings may have some in common with the so called Mozart-effect for music. In fact, previous studies found that listening to Mozart Sonata for two pianos in D major (K448) enhanced performance on spatial–temporal tasks (Hughes, 2001). It has motivated the scientific community in exploring the beneficial effects of musical stimuli on a variety of diseases over the past two decades (Hughes, 2001; Vinciguerra, 2017). The reported benefits of Mozart effect on neurophysiological activities include increased EEG power and coherence, increased correlations of neurophysiological activity on the left frontal and temporal areas, improved walking ability, and on neuropsychological abilities such as increased spatial–temporal reasoning after piano lessons in preschool children and improved IQ test results (Escher and Evéquoz, 1999; Hughes, 2001; Trappe and Voit, 2016; De Bartolo et al., 2020b).

Similarly, we named as Michelangelo effect the improvement of subjects’ performance in presence of an artistic masterpiece. This amelioration could be motivated by a general arousal of the brain, but more specifically, to the capacity of the beauty of art to activate specific brain areas, including sensorimotor ones, according to previous studies (Freedberg and Gallese, 2007; Umilta’ et al., 2012; Di Dio et al., 2016). However, further studies are needed to deeper investigate whether it acts as a priming effect for the successive motor performance or if it works in parallel to the cognitive process related to psychological aspects such as the perceived load demand of the task and the level of participation.

Many factors concur in making a painting a masterpiece: colors, lines, elements, style, shadows, details and the general resulting overview. In this study, the control stimuli were balanced for colors and brightness, but they were blurred paintings, with less details than the artistic stimuli. Furthermore, the artistic stimuli included in this study were very different from each other in terms of number of details (i.e., few in The Dance of Matisse or many in The Night Café of Van Gogh), the presence of human figures (i.e., none in The Bedroom of Van Gogh, up to the 13 people in the Rowers Breakfast of Renoir), the curvatures of tracts (i.e., curve pathways characterize The great wave of Kanagawa whereas linear ones The Three Musicians of Picasso), and many other perceptual factors. However, the main effect of the type of paint was not statistically significant for the three kinematic parameters reported in Table 1. A higher variability in these parameters was even recorded for control stimuli, as shown by the higher standard deviations recorded for them, leading to a significant interaction effect. However, further studies are needed to investigate which elements of a masterpiece may affect the performance of subjects, planning balanced comparisons of specific features such as previous investigations did for exploring the effects of artwork content on brain activations.

**TABLE 1:** Experiment 1: mean ± standard deviation of the time to complete the task (TCT), the length of hand Trajectory (LoT) and the root mean square of hand trajectory in axis orthogonal to canvas (RMSe).

**Parameters and statistics**		**TCT (s)**	**LoT (m)**	**RMSe (m)**
Mean Art ± standard deviation		5.40 ± 1.97	6.74 ± 1.59	0.08 ± 0.05
Mean Control ± standard deviation		5.51 ± 2.55	7.71 ± 3.54	0.08 ± 0.03
Main effect Art vs. Control	*F*(1,190)	0.257	12.268	2.476
	*p*	0.613	**0.001**	0.117
	ES	0.001	0.061	0.013
Main effect Type of paint	*F*(1,190)	1.581	0.328	0.813
	*p*	0.123	0.965	0.605
	ES	0.070	0.015	0.037
Interaction	*F*(9,190)	4.419	0.760	0.172
	*p*	**<0.001**	0.654	0.997
	ES	0.173	0.035	0.008

*Then the results of mixed analysis of variance: *F*-values (with degrees of freedom) and relevant *p*-values (in bold if statistically significant) and Effect Size (ES, computed as partial eta-squared).*

**TABLE 2:** Experiment 2: Baseline clinical assessment of patients mean ± standard deviation for the kinematic parameters (TCT: time to complete the task, LT: length of trajectory, RMSe: root mean square of erroneous movement orthogonal to the canvas) for the four Patients and relevant within-subject comparison.

**Patients, parameters and statistics**			**Patient 1**	**Patient 2**	**Patient 3**	**Patient 4**
Baseline Clinical Assessment	Gender		Male	Female	Male	Male
	Side of hemiparesis		Right	Left	Left	Left
	Type of stroke		Ischemic	Hemorrhagic	Hemorrhagic	Ischemic
	Fugl-Meyer total score		118	102	117	121
	Fugl-Meyer sensitivity		No deficit	No deficit	1 (arm)	No deficit
	Fugl-Meyer proprioception		1 (elbow)	No deficit	1 (elbow and wrist)	No deficit
	Box and Block test (blocks)		20	32	35	46
	9-hole peg test (s)		29.3	39.3	18.5	17.5

**Type of stimuli during virtual reality task**			**Art**	**Art**	**Control**	**Control**

Kinematic parameters recorded in the 1^st^ and 4^th^ sessions and relevant comparisons	TCT (s)	Session 1	17.6 ± 6.9	11.6 ± 1.9	7.7 ± 1.5	7.3 ± 2.5
		Session 4	7.0 ± 0.6	8.3 ± 0.9	7.3 ± 1.1	5.9 ± 1.4
		*p*-value	**0.008**	**<0.001**	0.465	0.125
	LoT (m)	Session 1	6.7 ± 1.5	7.8 ± 1.3	5.4 ± 0.7	7.6 ± 1.9
		Session 4	5.1 ± 0.3	6.2 ± 0.6	4.9 ± 0.4	8.4 ± 1.7
		*p*-value	**0.034**	**0.008**	**0.029**	0.385
	RMSe (m)	Session 1	0.10 ± 0.04	0.08 ± 0.03	0.11 ± 0.03	0.10 ± 0.02
		Session 4	0.05 ± 0.01	0.04 ± 0.01	0.10 ± 0.03	0.14 ± 0.04
		*p*-value	**0.041**	**0.007**	0.504	**0.022**

*p-values in bold if statistically significant (<0.05).*

Given the small sample size in the experiment two, the obtained results could be affected by the different clinical conditions of patients. For example, the patients treated with artistic stimuli showed worst performance at Box and Block test and 9-hole peg test and also longer TCT in the first sessions with respect to those treated with control stimuli. However, by observing LoT and RMSe values at the first session, we could compare the performance of patient 1 (art) with that of patient 3 (control), as well as that of patient 2 (art) with that of patient 4 (control). Patients treated with artistic stimuli (1 and 2) had a significant reduction in both these parameters. Conversely, patient 3 showed a reduction only in LoT, and patient 4 even showed a significant increment in RMSe.

It is important to mention the limits of the present study: first of all the limited sample size of patients enrolled in the experiment 2, then the age difference between subjects involved into the two experiments, the eventual bias related to our arbitrary selection of paintings (their styles, their contents, if landscape paintings or with human figures, and so on), the difference in details of these paintings and their modified control versions (we only balanced artistic and control stimuli for colors and brightness, but losing details in control stimuli), to absence of measures of physiological data of subjects during the task execution, and the absence of data about their artistic knowledge and/or sensitivity. At the same time, this study has also some strengths: first of all its novel approach to upper limb rehabilitation in patients with stroke using art-therapy combined with virtual reality, the high ecological validity of the motor task, the high engagement and motivation of patients, the robust measures of performance (based on kinematic, behavioral and self-reported assessment), and the promising results for patients with stroke and perhaps other neurological diseases. However further studies are necessary to confirm and extends these results, especially regarding the interpretation of the significant effect in the physical demand that deserves caution at the moment (given the *p*-value of 0.049).

This study was a first step for testing the usability of a VR system to allow subjects to interact with an art masterpiece, for analyzing if the artistic content could increase motivation and performance, and to compute the required sample size for designing a randomized controlled trial in which the Michelangelo effect can be tested if effective for improving the neurorehabilitation outcomes. This approach could open a novel way for rehabilitation program for multiple users and can be helpful in administering to neurological patients an art therapy for upper limb recovery.

## Data Availability Statement

The raw data supporting the conclusions of this article will be made available by the authors, without undue reservation.

## Ethics Statement

The studies involving human participants were reviewed and approved by Ethics Committee of the Santa Lucia Foundation. The patients/participants provided their written informed consent to participate in this study.

## Author Contributions

MI conceptualized the study. GM, MI, and GT wrote the first draft of this manuscript. GT and CC designed the 3D models and implemented the VR setup. MA, CC, and NC programmed the experimental tasks, collected the data, and computed the kinematic values. MI and FB performed all the statistical data analysis. GA, FM, and SP supervised the study. All authors revised the manuscript and provided important contributions.

## Conflict of Interest

The authors declare that the research was conducted in the absence of any commercial or financial relationships that could be construed as a potential conflict of interest.

## Supplementary Material

Supplemental Video 1 For an example of the experimental task can be seen at this link: https://youtu.be/Y4hP_t6bEH4.
